# H-MRS Correlates of Deep TMS in Schizophrenia: Insights From a Randomized Sham-Controlled Study on Negative Symptoms

**DOI:** 10.1192/bjo.2025.10168

**Published:** 2025-06-20

**Authors:** Gulesh Kumar, Nishant Goyal, Aniruddha Mukherjee

**Affiliations:** 1 St. Cadoc Hospital, Newport, United Kingdom; 2 Central Institute of Psychiatry, Ranchi, India

## Abstract

**Aims:** Negative symptoms of schizophrenia are disabling and often show inadequate response to antipsychotic treatment. Dysfunction in cortical regions such as the anterior cingulate cortex (ACC) and medial prefrontal cortex (mPFC) has been implicated in these symptoms. While repetitive transcranial magnetic stimulation (rTMS) has shown efficacy, deep transcranial magnetic stimulation (dTMS) offers the advantage of targeting deeper brain structures.

To assess the efficacy of high-frequency dTMS in improving negative symptoms of schizophrenia and to examine its effects as measured by proton magnetic resonance spectroscopy (h-MRS).

**Methods:** This sham-controlled, double-blind study randomized 46 patients with schizophrenia into active and sham dTMS groups. Participants received 10 sessions of high-frequency (10 Hz) dTMS at 100% of the resting motor threshold using an H7 coil over 2 weeks. Symptom severity was assessed using the Positive and Negative Syndrome for the Assessment of Negative Symptoms (SANS), and Clinical Global Impression (CGI) at baseline, 2 weeks, and 4 weeks post-treatment. h-MRS of the ACC and mPFC was performed at baseline and after 2 weeks of treatment.

**Results:** A total of 43 patients completed the study. While both groups showed improvement over time, the active dTMS group demonstrated significantly greater improvement in negative symptoms, as reflected by a reduction in SANS scores compared with the sham group (p=0.003) and improvement in the negative subscale of PANSS (p=0.044). h-MRS analysis revealed a positive correlation between ACC total N-acetylaspartate (tNAA) levels after 2 weeks of treatment and baseline SANS anhedonia subdomain scores.

**Conclusion:** High-frequency dTMS significantly improves negative symptoms and overall illness severity in schizophrenia. These findings highlight the potential role of dTMS as an adjunctive treatment and suggest that h-MRS may serve as a valuable biomarker for treatment response. Future studies with larger sample sizes are needed to further explore the therapeutic and neurobiological effects of dTMS.

